# Factors Associated With Post-traumatic Growth Among Healthcare Workers Who Experienced the Outbreak of MERS Virus in South Korea: A Mixed-Method Study

**DOI:** 10.3389/fpsyg.2021.541510

**Published:** 2021-04-22

**Authors:** Hye Sun Hyun, Mi Ja Kim, Jin Hyung Lee

**Affiliations:** ^1^Department of Nursing, Sangmyung University, Cheonan, South Korea; ^2^Department of Nursing Science, Daejeon Institute of Science and Technology, Daejeon, South Korea; ^3^Human IT Clinical Research Center, Chung-Ang University, Seoul, South Korea

**Keywords:** mixed methods, Middle East respiratory syndrome coronavirus, healthcare workers, post-traumatic stress disorder, post-traumatic growth

## Abstract

**Background:** Infectious disease outbreaks such as COVID-19 and MERS pose a major threat to healthcare workers' (HCWs) physical and mental health. Studies exploring the positive changes gained from adapting to traumatic events, known as post-traumatic growth (PTG), have attracted much attention. However, it is unclear which factors or experiences lead to PTG among HCWs. The purpose of this mixed-method study was to investigate factors associated with PTG among HCWs who experienced the MERS outbreak in South Korea, and fully describe their experience of developing PTG.

**Methods:** Quantitative data from 78 participants were collected using psychometric tools for Psychological distress, Resilience, and Support for coping, and Post-traumatic growth. Qualitative interviews were conducted with seven nurses. Data were analyzed using the qualitative content analysis method according to the sub-themes of resilience, which was the main factor associated with PTG.

**Results:** We found resilience to have a significant impact on PTG (ß =0.486, *p* = 0.001). Thus the qualitative interviews were analyzed using the core concepts of resilience. Qualitative interviews with nurses illustrated how participants experienced the development of resilience in terms of its sub-factors: hardiness, persistence, optimism, and support.

**Conclusion:** HCWs who endured the MERS outbreak showed high levels of PTG, and the analysis of the interview data provided a fuller understanding on the experience of remaining resilient and developing PTG. These results provide practical and pragmatic information helpful for developing intervention strategies and protocols that can help HCWs transform adversity into growth and development.

## Introduction

Numerous worldwide public health threats occurred in the 21st century, including the Severe Acute Respiratory Syndrome (SARS), Middle East Respiratory Syndrome Coronavirus (MERS), and 2019 novel Coronavirus (COVID-19). In June of 2015, an outbreak of MERS in South Korea resulted in 186 diagnoses (Korea Disease Control Prevention Agency, 2015). It was the biggest outbreak outside of middle-east, which revealed many vulnerable points in Korean biorisk management and medical system (Oh et al., 2015). Investigators identified the following as causes of the widespread: delayed diagnoses, undetected superspreaders, patients visiting multiple doctors for a second opinion, uncontrolled family visits, untruthful disclosure of information by patients, veiled government communication on outbreak status, and inadequate infection management system (Kim et al., 2017). Unfortunately, 97% of suspected MERS cases in Korea were hospital-acquired infections (Oh et al., 2015). Among the confirmed cases, 39 were healthcare workers (HCWs) (21% of total cases), consisted of 8 physicians and 15 nurses (Oh et al., 2015). These reports indicate that the outbreak was entirely due to hospital-acquired infection, reflecting the failure of the infection management system and administrative policy.

Infectious illnesses with high prevalence and mortality rates pose a significant threat to the psychological well-being of HCWs. Numerous studies reported high post-traumatic stress levels among HCWs who provided care for SARS patients (Chan and Huak, 2004; Chong et al., 2004; Chua et al., 2004; Maunder et al., 2004; Sim et al., 2004). Similarly, various studies also observed the negative psychological impact of MERS on HCWs (Jung et al., 2016, 2020; Kim and Choi, 2016). Researchers report that HCWs who provided direct care to MERS patients in S. Korea experienced PTSD symptoms (Jung et al., 2016), burnout, and fear (Jung et al., 2016; Kim and Choi, 2016; Jun et al., 2018; Seo et al., 2020), and turnover intention (Jung et al., 2020).

Looking at the surrounding circumstances during the MERS outbreak in Korea, one can easily suspect that Korean HCWs were exposed to the same or even higher levels of stress due to the absence of proper systemic support, policies, or guidelines (Kim et al., 2017; Kim, 2018). Hence, it is essential to identify the factors associated with the negative psychological impact of infectious diseases and develop effective coping strategies and supportive programs for future crisis (Buselli et al., 2020; Firew et al., 2020; Tan et al., 2020).

Prior studies have tended to assess the psychological effects, such as post-traumatic stress symptoms and depression, by investigating the differences between groups exposed and not exposed to outbreaks. Longitudinal studies have also investigated the long-term psychological impact of epidemics. Recently, researchers have been investigating the negative impact of COVID-19 on the physical and psychological health of HCWs (Buselli et al., 2020; Firew et al., 2020; Tan et al., 2020). Many of these investigators suggest the need to identify factors that can protect and support HCWs during the crisis, and frequently discussed concepts in similar inquiries are resilience and post-traumatic growth (Lancee et al., 2008; Siqveland et al., 2015; Xu et al., 2016; Albott et al., 2020; Yildirim et al., 2020).

Traumatic experiences can be detrimental, yet overcoming a trauma may lead to constructive outcomes. Several studies explored the positive changes gained from adapting to traumatic events and reported that the negative impact of trauma-related stress could be mediated by resilience or post-traumatic growth (PTG) (Lancee et al., 2008; Siqveland et al., 2015). Post-traumatic growth (PTG) is the positive psychological changes that emerge from experiencing life struggles or adverse events (Tedeschi et al., 2018). Researchers report that PTG can protect HCWs or ameliorate their psychological impact post-traumatic events (Lancee et al., 2008; Rodríguez-Rey et al., 2019; Okoli et al., 2021). It can lead to a reduction of psychological symptoms such as anxiety and depression (Willie et al., 2016; Aderhold et al., 2019) and improve quality of life among traumatized HCWs (Martz et al., 2018). Moreover, longitudinal studies concluded that PTG could last for years (Schubert et al., 2016; Husson et al., 2017), which is essential for HCWs who may be exposed to traumatic events repeatedly.

Many researchers identified resilience as one of the influential factors for PTG (Lancee et al., 2008; Yildirim et al., 2020). PTG is different from resilience in that PTG is an adaptive response, growth, and gain from traumatic events. In contrast, resilience is an individual characteristic or behavioral tool related to coping mechanisms (McCleary and Figley, 2017). Even though the relationship between resilience and PTG are well documented, it is unclear how resilience leads to PTG among trauma-exposed individuals. Consequently, we conducted a descriptive survey to investigate factors associated with PTG among HCWs who experienced the MERS outbreak in South Korea, and fully describe their experience of developing PTG via implementing a mixed-method approach.

Currently, frontline HCWs fighting COVID-19 are left exposed to trauma and burnout, and immediate measures are needed to protect them from adverse psychological effects (Albott et al., 2020; Firew et al., 2020). Echoing other researchers, we would argue that investigating factors associated with and experience of developing PTG can provide timely information for designing supportive strategies and protocols for promoting resilience and PTG among HCWs (Albott et al., 2020; Pollock et al., 2020), thus minimizing the detrimental impact of COVID-19.

Therefore, the purpose of this mixed method study was to investigate factors associated with PTG among HCWs who experienced the MERS outbreak in South Korea, and fully describe their experience to better understand the process of developing PTG. The proposed hypotheses for the quantitative phase of the study were: (1) The levels of psychological distress will have a meaningful relationship with the levels of PTG; (2) Resilience will have a significant impact on PTG; and (3) Support for coping will have a significant impact on PTG.

## Materials and Methods

### Study Design

This study used a sequential, explanatory mixed-method research design. The sequential collection of quantitative data on the factors associated with PTG in healthcare workers who experienced the MERS outbreak was followed by qualitative interviews. The purpose of this sequential design was to “use the qualitative strand to explain the quantitative results” (Creswell and Clark, 2011, p. 63). During the quantitative phase, a questionnaire was conducted to investigate levels of distress, resilience, support for coping, and PTG among HCWs with MERS experience. During the qualitative phase, data from seven individual interviews were analyzed using the qualitative content X analysis method to gain a perspective on the influential factors for PTG, perceived as significant by the participants.

### Participants

The current study was conducted in the Spring of 2016 at two major hospitals located in a large metropolitan area immediately after the last case of MERS infection in November of 2015. During the planning stage, we conducted a priori power analysis using G^*^Power 3.1.9.4 for sample size estimation using a linear multiple regression with fixed model (Hsieh et al., 1998), *R*^2^ deviation from zero with three predictors, α = 0.05, and power = 0.95. Due to the absence of identical studies, we set the effect size based on Cohen's universal rule of thumb for the medium effect size of 0.15 for multiple regression (Cohen, 1988), which is a common method of assessing effect size in allied health research (Kaldas et al., 2009; Elsworth and Osborne, 2017). However, the number of designated hospitals in the metropolitan area were limited, making the total number of potential participants only about 90. Since the relationship between resilience and PTG has been well-documented, we decided to proceed and aim for the highest number of participants.

Inclusion criteria for participants were HCWs (e.g., doctors, nurses, allied health care professionals, paramedics, nurse's assistants, support staff, etc.) who provided direct or indirect care for patients diagnosed with, or suspected of having MERS in these two hospitals (designated by the S. Korean government as a dedicated hospital for MERS). HCWs who provided direct care include physicians or nurses who had face-to-face contact with MERS patients during routine physical examinations, vital checks, sample collections, and transport. HCWs responsible for indirect care include pathologists, nursing assistants, and hospital workers who performed tasks such as lab testing, disinfecting, cleaning, etc. A total of 78 HCWs were recruited for the quantitative study; all subjects voluntarily participated in this study.

We used purposeful sampling to recruit participants for the qualitative phase (Creswell, 2014). We began by screening occupation groups with higher scores for PTG and those who provided direct person-to-person care to MERS patients in quarantine rooms. Because the nurse group showed the highest level of PTG, we sent out invitations to nurses for voluntary participation. Of those, seven nurses agreed to participate in the in-depth interviews. The decision to select nurses with higher PTG scores was based on the assumption that interviewing a higher PTG group would provide us with fuller and richer data, leading to a better understanding of their experience of developing PTG.

### Measurements

We measured levels of event-specific distress, resilience, PTG, and support for coping as well as documenting the participants' demographic information on population characteristics and their personal experiences with and exposure to MERS.

#### Psychological Distress

Psychological distress was measured using the Korean version of the Impact of Event Scale-revised (IES-R-K), initially developed by Weiss and Marmar (1997) and translated by Eun et al. (2005). This questionnaire consists of 22 items that evaluate the frequency of related symptoms from the past week, using a five-point Likert-type scale. The accumulated results range from 0 to 75; they include six items on hyperarousal, six on avoidance, five on intrusion, and five on sleep and numbness. A score of 17 or lower is considered normal. People with scores of 18 or above are at high risk for PTSD (Eun et al., 2005). IES-R-K has been established as a reliable tool to assess PTSD symptoms with both good reliability and validity (Lim et al., 2009). The Cronbach's alpha was 0.70 for avoidance, 0.87 for hyperarousal, and 0.63 for intrusion upon translation (Eun et al., 2005). In this study, the overall Cronbach's alpha was 0.95, 0.85 for hyperarousal, 0.94 for avoidance, 0.88 for intrusion, and 0.54 for sleep and numbness.

#### Resilience

The Korean version of the Connor–Davidson Resilience Scale (K-CD-RISC) (Baek et al., 2010), developed by Connor and Davidson (2003), was used to measure participants' levels of resilience. The K-CD-RISC includes nine items on “hardiness,” eight on “persistence,” four on “optimism,” two on “support,” and two on “spiritual attributes.” Participants use a five-point Likert-type scale, rating each item from 0 for “not true at all” to 4 for “true nearly all the time.” K-CD-RISC is widely utilized to measure resilience among Korean subjects with well-established reliability and validity (Baek et al., 2010). The Cronbach's alpha for K-CD-RISC in this study was 0.94.

#### Support for Coping

We used the subcomponent on coping from the questionnaire on Changes in Life's Priorities and Coping (Chan and Huak, 2004) to measure support for coping. This tool was initially developed to measure the impact of the SARS outbreak. The scale consists of nine items, rated using a six-point scale from 1 (strongly disagree) to 6 (strongly agree). The questions focus on “circumstances that contribute to coping.” Because these circumstances essentially describe helpful support strategies and systems, the authors describe them as “support for coping with MERS.” In this study, the Cronbach's alpha was 0.85, but the tool has not been formally studied for its reliability and validity.

#### Post-traumatic Growth

To measure PTG, we used the Korean version of the Post-Traumatic Growth Inventory (K-PTGI), initially developed by Tedeschi and Calhoun (1996) and translated and validated by Song et al. (2009). The K-PTGI includes six items on “changes of self-perception,” 5 on “increase in interpersonal depth,” 3 on “finding new possibilities,” and 2 on “increase in spiritual interest,” rated using a 6-point Likert-type scale. The scale ranges from 0 for “experienced no change” to 5 for “experienced a great degree of change.” The scores ranged from 0 to 80, with higher scores indicating greater levels of growth. K-PTGI has been established as a valid tool for studying PTG of Korean subjects with good reliability and validity (Song et al., 2009). The Cronbach's alpha for the inventory was 0.92 on development and 0.95 overall for this study.

### Data Collection

We designed a cross-sectional survey and distributed it to HCWs who provided direct or indirect care to MERS patients at two designated hospitals from February to March of 2016. A total of 78 HCWs participated in the self-report questionnaire, which took ~20 min to complete.

For the qualitative phase, the second author conducted in-depth interviews with participants who provided additional written consent to participate and be audio-recorded. They were fully informed of the purpose and method of the interviews, including their rights as a research participant. Everyone accepted the interview invitation without refusal. Participants were recruited until thematic saturation was reached (Guest et al., 2006). Because we set the dimensions of qualitative data based on the subfactors of resilience, we considered a thematic saturation achieved when responses from the participants' began to show a redundancy and ceased to generate novel concepts relevant to existing factors. Interviews lasted ~40 min on average and were conducted in the nurses' rest area behind closed doors to ensure a safe and quiet environment. First, the interviews started with general questions about their brief background as a nurse and assigned duties. A set of open-ended questions for a semi-structured interview were prepared in advance (see Table 1), but the interviewer adapted and devised new but relevant questions as the interviews progressed. To be more comprehensive, the researchers devised questions related to the following categories: personal experiences, professional experiences, particular behaviors, own values and opinions, relevant knowledge, and sensory experience related questions. See Table 1 for sample questions used during the interviews.

**Table 1:** Example of open-ended questions for the semi-structured interviews.

**Sample questions**
How did you feel about having MERs patients under your direct care?
How did your friends or family react when they heard about you working in the quarantine unit?
What do you vividly remember from those days? Any scenes, smells, sounds, etc?
What were the most difficult aspects of your work as a HCW during the outbreak?
How did you manage to cope with those difficulties?
Was there anything that helped you during those moments?
How did your knowledge about caring for patients and epidemic illness change?
Was there any systemic or governmental support that you found helpful?
Have you observed any changes in yourself having been through the outbreak?
As a HCW, do you feel that you have changed from having the MERs experience?
Were there any special moments, talks, encounters that you value from those times?

To probe their experience of overcoming traumatic experiences, the interviewer asked about their personal experience of MERS, their reaction to and emotional responses to working with MERS patients, and the coping strategies or factors associated with overcoming related issues. The interviewer engaged in attentive listening and reflecting, probing with additional questions to clear up any potential misinterpretations or confusion.

To improve the credibility of the qualitative data, we conducted member checking as a method of triangulation by asking the participants to review the full scripts of the interviews and provided chances to elaborate further as needed.

### Data Analysis

The quantitative data were analyzed using SPSS 20 statistical software. First, we conducted descriptive statistics, a *t*-test, and ANOVA with the Scheffé *post-hoc* test to investigate whether the participants' characteristics were associated with any differences in the PTG scores. Then, we performed a hierarchical multiple regression analysis to identify factors influencing PTG, adjusting for covariates that showed a significant difference in the *t*- and *F*-test.

For the qualitative phase, we interviewed the occupation group with the highest PTG scores on their experience of living through MERS. To gain practical insight on the experience of developing PTG, we utilized the deductive content analysis approach delineated by Elo and Kyngäs (2008) to check the current data against existing data in a new context (Catanzaro, 1988). For this, we developed a categorization matrix and operational definitions based on the sub-factors of the psychometric tools that were found to have a significant impact on PTG.

We followed the following steps to analyze the verbatim interview data. Investigators conducted multiple readings of the texts, analyzed them line by line to generate codes that best represented each meaning unit to their context. These codes were checked against pre-existing codes to group them into sub-themes. The purpose of this process was to obtain a “condensed and broad description of the phenomenon” (Elo and Kyngäs, 2008, p. 108). Two investigators took the steps above independently, then compared the codes and sub-themes against each other to discuss and arrive at an agreement. The third investigator verified the codes and sub-themes generated. Finally, the sub-themes were grouped into categories and compared against the categorization matrix and operational definitions of sub-factors of the resilience that significantly impacted PTG. For this, we used the operational definitions from the Korean version of the Connor–Davidson Resilience Scale (see **Table 7**) as the categorization matrix to match the identified sub-themes and categories (Elo and Kyngäs, 2008) to the corresponding subfactors of resilience.

### Ethical Considerations

The study was reviewed and approved by the Sangmyung University Institutional Review Board (SMUIRB-ex-2015-001). During the consent process, we explained the nature of the study and the voluntary nature of participation. Participants were informed of their right to withdraw at any time without reprisal. Researchers described the anticipated benefits and potential risks of the study, as well as the confidentiality of responses. All participants provided informed consent before data collection, and all data were collected anonymously to respect the participants' privacy.

## Results

### Quantitative Results

#### The Sociodemographic Characteristics of the Participants

Table 2 shows the general characteristics of the 78 participants. The participants included nurses (73.1%), physicians (6.4%), and other health workers (20.5%). The mean age was 35.2 (SD = 9.19) and most were female (82.1%). During the outbreak, 66.7% provided direct person-to-person care to MERS patients, and 52.6% indicated that they or their family members were exposed to MERS. Four participants (5.1%) were diagnosed with MERS, and five (6.4%) were placed in quarantine. Following the outbreak, 11.5% felt the need for psychological care from a professional, but none received such care (see Table 2).

**Table 2:** General characteristics of participants (*N* = 78).

**Variables**	**Categories**	***n***	**%**
Age	<35	42	53.8
	35–49	29	37.2
	≥50	7	9.0
Gender	Male	14	17.9
	Female	64	82.1
Level of education	≤High school	4	5.1
	College or University	56	71.8
	≥Master's degree	18	23.1
Have religion	Yes	37	47.4
	No	41	52.6
Marital status	Single	45	57.7
	Married	33	42.3
Work schedule	Only day time	36	46.2
	Shift work	42	53.8
Occupation	Doctor	5	6.4
	Nurse	57	73.1
	Other health worker	16	20.5
Direct contact with MERS patients	Yes	52	66.7
	No	26	33.3
Personal exposure to MERS	Yes	41	52.6
	No	37	47.4
Have been diagnosed with MERS	Yes	4	5.1
	No	74	94.9
Placed under quarantine due to MERS	Yes	5	6.4
	No	73	93.6
Other trauma exposure	Yes	10	12.8
	No	68	87.2
Wanted psychological service	Yes	9	11.5
	No	69	88.5
Have used psychological service	Yes	0	0.0
	No	78	100.0
Received service award	Yes	51	65.4
	No	27	34.6

#### Participants' Psychological Distress, Resilience, Changes in Life Priorities, Coping, and Post-traumatic Growth

The mean score for psychological distress (IES-K) was 10.09 (SD = 11.57). We recorded participants with scores of 18 or above as it indicates a high risk for PTSD (Eun et al., 2005), and found that 16.7% of the participants had scores equal to or higher than 18. The analysis results on the primary variables are listed in Table 3.

**Table 3:** Psychological distress, resilience, and support for coping with MERS and posttraumatic growth of participants.

**Variables**	**Respondents (*****N*** **=** **78)**	
	**Mean**	**SD**
Psychological distress (total)	10.1	11.57
Hyper-arousal	1.8	2.84
Avoidance	2.6	4.59
Intrusion	3.5	3.33
Sleep and numbness	2.1	1.96
High risk group *n*(%)	13(16.7%)	
Normal group	65(83.3%)	
Resilience (total)	64.7	14.96
Hardiness	21.9	6.30
Persistence	18.7	4.44
Optimism	10.3	3.36
Support	6.2	1.25
Spiritual in nature	4.6	1.62
Support for coping with MERS	4.4	0.92
Posttraumatic growth (total)	29.2	18.54
Changes of self-perception	15.9	4.32
Increase of interpersonal depth	2.5	3.57
Finding new possibilities	9.2	2.00
Increase of spiritual interest	4.4	1.53

#### Relationship Between the Participants' Levels of Psychological Distress, Resilience, Support for Coping With MERS, and PTG

Correlation analysis of PTG scores showed statistically significant small to moderate correlations with resilience (*r* = 0.422, *p* < 0.001) and support for coping with MERS (*r* = 0.326, *p* = 0.004), but it did not indicate a significant relationship between PTG score and psychological distress (see Table 4).

**Table 4:** Correlations among psychological distress, resilience, support for coping with MERS and PTG (*N* = 78).

**Variables**	**Psychological distress**	**Support for coping with MERS**	**Resilience**	**PTG**
Psychological distress	1	−0.101	−0.304**	−0.047
Support for coping with MERS		1	0.351**	0.326**
Resilience			1	0.422**
PTG				1

** *P < 0.01, PTG, posttraumatic growth*.

#### Factors Influencing Post-traumatic Growth

Regression analysis was performed to identify significant predictors for PTG. Because the Durbine–Watson value was 2.172, we assumed that there was no auto-correlation problem. The tolerance level was 0.797–0.972 and the maximum Variance Inflation Factor (VIF) was low at 1.255, showing no problem with collinearity.

Among the participants' general characteristics, “type of occupation” was one factor associated with a significant difference in PTG scores (*F* = 3.918, *p* = 0.024; See Table 5). To test the confounding effects of occupation type, a hierarchical multiple regression analysis was performed. The first model utilizing occupation type as a dummy variable, produced *R* square of 0.07 (*F* = 3.918, *p* = 0.024).

**Table 5:** Comparison of PTG according to participant characteristics (*N* = 78).

**Variables**	**Categories**	**Mean**	**SD**	***t*/*F***	***p***
Age	<35	26.57	17.69	1.381	0.258
	35–49	30.97	18.53		
	≥50	38.14	22.70		
Gender	Male	24.29	21.44	−1.106	0.272
	Female	30.33	17.85		
Level of education	≤High school	39.25	17.50	0.640	0.530
	College or University	28.39	17.35		
	≥Master's degree	29.67	22.45		
Have religion	Yes	30.11	19.73	0.389	0.698
	No	28.46	17.61		
Marital status	Single	26.27	16.41	−1.675	0.098
	Married	33.30	20.68		
Work schedule	Only day time	29.56	19.52	−0.137	0.892
	Shift work	28.98	17.90		
Occupation	Doctor (a)	7.60	8.76	3.918	0.024^†^ (a < b,c)
	Nurse (b)	30.63	18.83		
	Other health care worker (c)	31.06	15.93		
Direct contact with MERS patients	Yes	31.67	19.12	1.655	0.102
	No	24.38	16.63		
Personal exposure to MERS	Yes	31.67	19.12	1.052	0.296
	No	24.38	16.63		
Have been diagnosed with MERS	Yes	23.50	20.04	0.696	0.489
	No	30.13	18.46		
Placed under quarantine due to MERS	Yes	24.00	18.06	0.849	0.399
	No	29.60	18.64		
Other trauma exposure	Yes	33.90	21.15	0.849	0.399
	No	28.56	18.20		
Wanted psychological service	Yes	25.22	24.21	−0.689	0.493
	No	29.77	17.83		
Received service award	Yes	27.63	17.60	−1.059	0.293
	No	32.30	20.20		

† *Result from the Scheffé post-hoc test*.

Although a meaningful relationship between the levels of psychological distress and PTG scores was not observed in this study, we included psychological distress as one of the independent variables for the regression analysis as previous studies have confirmed this relationship (Sattler et al., 2014; Siqveland et al., 2015). Thus, the second model inputting all the variables revealed that 26.8% of the total variance in PTG can be explained by occupation type, psychological distress, resilience, and support for coping with MERS (*F* = 6.638, *p* < 0.001). Notably, the analysis revealed that resilience (ß = 0.392, *p* = 0.001) was the only significant predictor of PTG, over and above the other variables. The outcomes of these analyses are as shown in Table 6.

**Table 6:** Regression model of PTG.

**Variables**	***B***	***SE***	**β**	***t***	***p***	**Durbin–Watson**	**Adjusted *R*^**2**^**
Constant	−19.186	11.058		−1.735	0.087	2.172	0.268
Occupations dummy1 (doctor)	−23.366	7.439	−0.311	−3.141	0.002		
Occupations dummy2 (others)	−1.089	4.538	−0.024	−0.240	0.811		
Support for coping with MERS	3.809	2.110	0.188	1.805	0.075		
Psychological distress	0.192	0.166	0.120	1.159	0.250		
Resilience	0.486	0.135	0.392	3.589	0.001		

F_(5,72)_ = 6.638, p < 0.001. Reference group: Occupations *nurse.

### Qualitative Results: Content Analysis

#### Sociodemographic Characteristics of the Participants

A total of seven nurses who had direct person-to-person care experience for MERS patients participated in the qualitative phase. All were female, and three were married; they ranged in age from 25 to 47. They had 2–4 years of experience in infection units and 2–23 years of the overall experience at the time of the recruitment. All seven had a bachelor's degree in nursing; six were staff nurses, and one was a charge nurse.

#### Findings

As the regression analysis revealed a significant impact of resilience on PTG, the interview data were analyzed according to the sub-factors of resilience. We postulated that such analysis would guide a better understanding of the experience of developing PTG. The Korean version of the Connor–Davidson Resilience Scale maintained good reliability and validity. Still, it needed reclassification of items to form a four-factor structure instead of five in the original scale and reconceptualization of the definitions to address the cultural differences (Baek et al., 2010). Therefore, the researchers laid out operational definitions of the sub-factors of resilience based on the validity study conducted by Baek et al. (2010) (see Table 7). Based on these operational definitions, the sub-themes were grouped and compared against the sub-factors of resilience: “hardiness,” “persistence,” “optimism,” and “support” to identify corresponding concepts that may be significant for promoting PTG.

**Table 7:** Operational definitions of the sub-factors of the Korean version of Connor–Davidson Resilience Scale (Baek et al., 2010).

**Sub-factors**	**Operational definitions**
Hardiness	One is emotionally and mentally stable under stressful and unfortunate circumstances with a strong will or conviction to withstand the adversity
Persistence	One is able to tolerate negative affect, engage in “circumspect thinking” and make decisions despite the stressful situation
Optimism	One remains hopeful regarding the future and anticipates positive outcome of a specific issue
Support	One is able to receive help from other people and maintains close and meaningful relationships

The qualitative analysis revealed 12 sub-themes and 38 codes that describe the participants' experience relevant to the formation of resilience. Upon a careful comparison of each sub-themes to the sub-factors of resilience, the investigators attributed two sub-themes to “hardiness,” three to “persistence,” three to “optimism,” and finally four to the theme of “support” (see Table 8).

**Table 8:** Sub-themes and codes from the qualitative analysis.

**Theme**	**Sub-theme**	**Codes**
Hardiness	Accepting the responsibility	Accepting roles that come with the job
		Doing the best, even risking own lives
		Taking it as the ultimate responsibility
		Volunteering to work even during off-hours
	Enduring to fulfill the job	Handling even excessive requests
		Managing excessive workload
		Striving to save dying patients
Persistence	Becoming more competent	Adapting to the expanding and changing infection control guidelines
		Becoming familiar with wearing the PPE
	Enduring Negative Emotions	Battling fear from the seriousness of the infection
		Enduring stress from extra work
		Enduring the desire to quit
		Enduring the feeling of helplessness
		Enduring the separation from family and friends
		Feeling distressed from being suspected of carrying MERs
		Feeling unable to deal with inner issues
		Fighting fear from the uncertainty
		Tolerating negative emotions expressed by patients and doing the job
	Reframing thoughts about the situation	Feeling relieved for being able to depend on the PPE
		Realizing that staying in the hospital is the best way to protect self and loved ones
		Realizing the power of working together
Optimism	Acknowledging enhanced professionalism	Feeling more capable of caring for future patients
	Taking different perspectives	Becoming more appreciative of life
		Having a different perspective about the job
		Reframing it as highly meaningful and valuable life experience
	Taking pride in the experience	Feeling pride in the profession
		Feeling proud of having the experience
Support	Developing strong collegial relationship	Becoming more respectful of co-workers
		Developing intimate, trusting relationships
		Fighting for life together just like war combatant
	Exchanging help with colleagues	Processing issues and difficulties with colleagues.
		Sharing colleagues' burdens and responsibilities
	Receiving support from personal relationships	Husband insisting to stay together despite the danger
		Parents remaining supportive not showing all the worries
	Receiving systemic support	Having updated infection control manual and better equipment
		Receiving education and training for infection control
		Receiving support and acknowledgment from supervisors
		Receiving temporary staff from the departments

##### Hardiness

Hardiness was defined as “emotionally and mentally stable under stressful and unfortunate circumstances with a strong will or conviction to withstand the adversity.” Participants endured the adverse circumstances by “Accepting the responsibility” and “Enduring to fulfill the job.” First, participants accepted the adverse situation by simply taking it as another part of their job as they stated, “Patients come and go, and I gotta do what I gotta do” and “I just had to do it no matter what.” One participant volunteered even during her off-hours: “I was off work (when the outbreak started) and I got a notification seeking volunteers. I simply thought, ‘I live nearby and I should help’.” Two of the participants focused on their sense of obligations as they stated: “My professional sense of obligation exceeded the desire to quit” and “I am a nurse and it is the ultimate job of a nurse to take care of ill patients.” This sense of professionalism drove them to perform even in apparent danger as one mentioned: “We risked our lives and fought MERS. We worked with the mindset of a soldier going into a battle.”

“Enduring to fulfill the job” was another aspect of hardiness in which they performed their best to save dying patients as one participant recalled, “For patients facing imminent death, I had to put all my effort to help them.” Sometimes, they had to endure even excessive requests from patients, as one participant stated:

One patient told me to go out and buy a mango juice, and another patient threw a bottle of water at me and yelled, “I cannot drink anything other than Evian!” Patients were unstable in severe distress, and I accepted that as a natural response as they were suddenly locked inside a quarantine unit.

##### Persistence

Persistence was defined as how the participants “tolerated negative affect, engaged in ‘circumspect thinking’ and made decisions despite the stressful situation.” In these aspects, we monitored how the participants persevered and coped through difficult times. The analysis yielded the following sub-themes: “Enduring negative emotions,” “Reframing thoughts about the situation,” and “Becoming more competent.”

From a professional aspect, participants had to endure negative emotions expressed by patients as one participant recalled:

Since patients were separated from their families and having a hard time, they openly expressed how frustrated and upset they were, and it was difficult for me. But when patients were not doing well and were at the end stage of life, we, the nurses, had to tolerate all their anger.

From a personal aspect, participants endured extended separation from their loved ones as one participant disclosed:

Not seeing my children was hard, but I endured. When I missed them too much, I asked my mom to bring them to a playground, and I watched them from a distance. My heart broke.

Reframing thoughts about the situation was one of the active strategies that helped participants endure the negative emotions and hardships associated with the outbreaks. At first, participants complained about having to deal with the uncomfortable protective gears, but they realized the importance of them and said:

As soon as I wore the gear, things appeared dark and foggy and I was sweating immediately. My hands felt like being tied up and I couldn't stand wearing it for more than 5 min. But it was the only thing that I could rely on. Actually, it was a relief that I had access to protective equipment.

Before MERS, I considered it a hassle to try on the basic protective gear, but now my attitude has totally shifted. It saved our lives.

One of the most difficult parts was dealing with separation from loved ones. However, participants were able to reassess this situation as they stated:

Staying separated from my family may be a sacrifice on my part, but I had a responsibility to protect them.

You never know what might happen to the community because of me. Of course, I wanted to see my friends and family. but I couldn't because of the potential danger I pose. I had to endure the sacrifice.

As the participants withstood the challenges, they became aware that they were better able to handle the situation more competently as they expressed:

To prevent further infections, we had to follow the infection control manual, although it kept changing. Otherwise, the consequences were scary. When we were wearing PPE, we became highly vigilant. Same for when we were taking them off, as we were worried we might transfer the virus.

Because of so much repetition, I got used to wearing the protective gear. Now, I can do it quickly without sweating a bit.

##### Optimism

The theme of “Optimism” was defined as how the participants “remain hopeful regarding the future and anticipate a positive outcome of a specific issue.” Through the experience, participants became more optimistic as they were able to “Take different perspectives,” “Acknowledge enhanced professionalism,” and “Take pride in the experience.”

In terms of taking different perspectives, participants developed a different perspective about their job and considered the MERS outbreak a valuable life experience as participants pointed out: “I realized that wearing the usual protective gown is nothing!” and “As I look back, it became a part of good memories after a while. It was a good experience!”

One of the most positive attitudes toward the MERS experience was associated with how they perceived it as an experience that enhanced their professionalism. Participants acknowledged that:

Now, I think there is no case I cannot manage well as I experienced the extreme worst cases. When I see severe patients now, I tell myself, “I survived it (MERS), so this is nothing!”

Because of the experience, I can handle future incidences like this without a desire to quit due to infectious diseases.

In fact, participants reflected on the MERS experience as a valuable experience and went further by taking pride in having the experience. Participants expressed how they were proud of their performance and achievement during the outbreak by saying that:

It is pride from the fact that I protected my patients under such circumstances. Though it was difficult, working together with my colleagues was fun and will remain a good memory.

I believe nurses did so much during the outbreak. Who could have done that if it wasn't for us (nurses)! We all stayed and treated all the patients well. Particularly, young nurses were excellent as they remained calm, firm, and kind.

Lastly, participants remembered the experience as something highly unique and extraordinary as they made the following remarks:

When everything was over, the hospital created and played a video to commemorate how we controlled MERS, I became deeply emotional. That process of going through MERS is still very vivid to us. Although it was tough, now it doesn't feel as intense as before, and it left us a good memory.

(Because of MERS) I experienced something that others haven't. It is pride.

##### Support

“Support” was defined as the participants' ability to “receive help from other people and maintain close and meaningful relationships.” Sub-themes relevant to “support” were: “Receiving systemic support,” “Receiving support from personal relationships,” “Exchanging help with colleagues,” and “Developing strong collegial relationship.” One participant recalled how receiving education and training for infection control was helpful by saying that:

As time passed, we received manuals for infection control and saw stabilization of the management system at both institutional and governmental levels. More specifically, hospitals replaced the protective equipment, and people from the Center for Infection Control visited more often and supervised us.

The support participants received from their loved ones were also highly significant. Participants stated:

It could have been worse if it was obvious that my parents were worried sick. Instead, they remained calm and supported me, and it helped me a great deal.

My husband helped me immensely. Although I could not see my children, he was right beside me and supported me throughout. He even said if the worst happens, he would rather die with me.

Notably, the most number of subthemes were attributed to their professional and collegial relationships. First, participants had the experience of exchanging help with colleagues as they recalled:

Because we can't vent anger to our patients whatever they do, we(colleagues) would talk things out with each other as we knew each other so well.

Frankly, it was hard for everyone. But we would take care of each other by consoling each other, bringing water, and volunteering to go in before others.

Furthermore, exchanging help was not limited to interdepartmental staff, as one participant specified:

At first, ICU nurses re-positioned to our unit avoided going into the quarantine. As days passed and they saw how we were struggling, their attitudes changed and they also came forward and worked with us side by side.

As a result, participants experienced shaping strong collegial relationships with their fellow nurses, and this was one of the most significant sources of support. Participants echoed this by saying: “I wasn't worried much as I was with my colleagues,” “I really liked my colleagues. We advocated for each other, worked with devotion. We endured because of each other;” and “As I did everything together with my colleagues, I began to adapt and depend on them.”

This collegial relationship developed further and became something extraordinary as one participant expressed, “What we had was beyond friendship as we shared intimate feelings.” In the same context, one participant claimed:

It was beyond a working alliance as we were in a life and death situation. Doctors and nurses from other hospitals were admitted with a confirmed MERS diagnosis, and it made us develop even a tighter relationship.

One participant emphasized that working through this ordeal together made them respect each other by saying that “We developed a mutually respecting relationship between senior and junior nurses and that wonderful relationship is still ongoing.” One participant considered this collegial relationship so special that she expressed: “Because we were putting our lives on the line, comradeship is a better word for it. We were telling each other, ‘I will go in, you rest!’”

## Discussion

This study employed a sequential explanatory mixed-method research design to explore the factors associated with PTG among healthcare workers who experienced the MERS outbreak. Based on the findings, we raise the following points for discussion.

### Discussion on the Quantitative Result

First, previous studies have reported that individuals who experience traumatic events, such as natural disasters or deadly infectious diseases, often show high levels of psychological distress related to PTSD (Chan and Huak, 2004; Lancee et al., 2008; Siqveland et al., 2015) and that such events have a negative impact on people's quality-of-life (Siqveland et al., 2015). In this study, 16.6% of participants were in the high-risk group for developing PTSD. This result confirms the findings of a study by Chan and Huak (2004). They reported that 20% of nurses and doctors showed signs of PTSD after the Severe Acute Respiratory Syndrome (SARS) outbreak in Singapore. Although outcomes may differ due to differences in the psychometric tools used, the current study also repeats the results of another study, which showed that 18–57% of healthcare workers in Toronto continued to show signs of PTSD or emotional distress even 1–2 years after the SARS outbreak. Studies suggest that the spread of a serious epidemic illness can endanger the mental health of healthcare workers. Consequently, we reiterate the recommendation that policymakers and public health officials must reinforce precautionary measures to prepare for future epidemic outbreaks (Wu et al., 2008; Buselli et al., 2020; Firew et al., 2020).

In this s**t**udy, participants' psychological stress levels did not show a significant relationship with the PTG levels, which contradicts the results from couple studies that found a meaningful relationship between psychological stress and PTG (Sattler et al., 2014; Siqveland et al., 2015). In the same line, previous studies investigating this relationship also yielded heterogeneous outcomes. Researchers who investigated trauma-exposed firefighters found that the degree of post-traumatic stress was inversely related to PTG, meaning that levels of PTG increased when post-traumatic stress decreased (Sattler et al., 2014). In a study involving Norwegian adults who experienced the 2004 tsunami in Thailand, PTG was a moderating factor that reduced PTSD's negative impact of PTSD on quality-of-life issues (Siqveland et al., 2015). These contrasting results may reflect certain characteristics of the participants or the tools used to measure psychological distress; such differences may prevent researchers from making a clear pronouncement. Further studies are therefore needed to investigate the relationship between PTSD and PTG in order to reach a reliable conclusion. However, the results of the present study are still meaningful because they show that levels of PTSD do not play a conclusive role in determining levels of PTG.

### Resilience, as a Significant Factor for Developing PTG

In this study, participants' level of resilience had a meaningful impact on their levels of PTG. Even though this relationship between resilience and PTG was not supported in a study conducted with HCWs working in Pediatric Intensive Care Unit (Rodríguez-Rey et al., 2017), multiple studies report a significant relationship between resilience and PTG, particularly in trauma-exposed HCWs, and illustrate how resilience directly influence the process of post-traumatic change (Oginska-Bulik and Kobylarczyk, 2015; Kang et al., 2018; Ogińska-Bulik and Zadworna-Cieślak, 2018). One possible explanation for this relationship is that HCWs who have a high level of resilience have a better chance of taking the secondary trauma as a challenge to overcome. This way of interpreting professional hardships puts one in a better position to handle stress, which in turn, promotes resilience. Moreover, people with a higher level of resilience tend to discover positive aspects of their traumatic experiences (Rodríguez-Rey et al., 2017; Kang et al., 2018; Yildirim et al., 2020). In addition, a study reported that those who are more determined and persistent in action are more likely to develop PTG, particularly of self-perception (Ogińska-Bulik and Zadworna-Cieślak, 2018). Hence, it can be postulated that the participants overcame the highly stressful events through resilience, which helped them perceive trauma as another challenge to overcome and recognize beneficial aspects of the experience, consequently leading to PTG.

Another explanation for this is that more resilient people have better access to inner resources related to self-perception and relationships. In terms of self-perception, they can better recognize changes in self-perception, such as developing a more optimistic and appreciative attitude or becoming aware of their strength. In terms of relationships, they have a stronger sense of closeness, acknowledge the necessity of relationships, and can depend on others when in need. A study found that resilient people are raised by supportive family, are able to deal with stressful events effectively, often possess psychological resources such as life satisfaction, optimism and tranquility, and are more likely to develop PTG (Zhai et al., 2015). This direct relationship between resilience and PTG was again recorded in this study. Because the characteristics of resilient people often discussed in the literature address the aspects of the implementation of inner-resources and resourceful relationships, the following section discusses the main content of the qualitative analyses related to sub-factors of resilience.

### Analysis Based on the Sub-factors of Resilience: Core of Hardiness

Sense of professionalism was at the core of “hardiness” that enabled participants to sustain themselves during stressful times. Sense of professionalism is directly related to the core professional nursing principles: human dignity, integrity, altruism, and justice (Schmidt and McArthur, 2018). Altruism is implied with the emphasis on compassion and the primacy of the patient's interests over personal interests (Schmidt and McArthur, 2018). In this study, it was evident that participants did not question or debate what tasks were within their job descriptions and undertook many tasks beyond their routine duties. Interestingly, “taking it as undeniable responsibility” was at the center of what helped the participants endure stressful situations. This concept “proving care to highly infectious patients as an unquestionable duty of nurses” was also echoed in another qualitative study (Kim, 2018). The nurses endured and handled innumerable tasks and requests to save their patients even though they were up against an epidemic illness without a clear management manual. Additionally, their act of hardiness was manifested in their demonstration of the will to fulfill their responsibility as they volunteered to support other nurses regardless of their assigned department (Schmidt and McArthur, 2018).

### Analysis Based on the Sub-factors of Resilience: Core of Persistence

Just as one of the factors of persistence was “tolerating negative affect,” participants in this study also reported living through various negative emotions. Upon analysis of the data, the core of these negative emotions appeared to be fear: fear from uncertainty, fear of losing patients, fear of infecting loved ones. Even though this fear evoked from witnessing patients' death and being separated from family was intense, participants showed how they addressed their fear with proper systematic support and psychological coping strategies. Among various strategies that they utilized to cope with this fear, participants were able to persist through reframing their perception about the situation and gaining better competency in handling the situation. In the same line, cognitive reframing has been identified as one of the main strategies nurses implement to build resilience (Hart et al., 2014). The role of cognitive reframing was further elaborated that it helps nurses to “to review and retrace their internal and external environments enabling them to promote psychological flexibility and adaptability” (p. 727). Hence, we can observe how the participants' cognitive reframing based on professional responsibility persisted the nurses through turmoil and crisis, leading to the development of full-blown resilience.

### Analysis Based on the Sub-factors of Resilience: Core of Optimism

As researchers echo that resilient nurses have a general sense of optimism and hopefulness (Hodges et al., 2008; Hart et al., 2014), we sought to determine the themes related to optimistic and hopeful outlook in the participants' interviews. What was unique to this particular group of nurses was that they attributed special meaning in their “action of caring and saving lives,” and even cultivated an optimistic outlook about the future outbreak. Eventually, this experience became something they felt proud of and something that enhanced their professionalism. The fact that the participants were no longer worried about their performance in future outbreaks speaks for their post-traumatic growth in both professional and personal aspects.

### Analysis Based on the Sub-factors of Resilience: Support

Just as resilience entails “support from others” as one of the sub-factors, participants also reported exchanging support from significant relationships. However, what was unique about their support was that the main source of relational support was from other nurses who endured the MERS outbreak together, not their friends and family members, because they were involuntarily separated from personal relationships. This separation from personal relationships did not stop them from exchanging social support; instead, they developed a strong and healthy collegial relationship even to the point that they would volunteer to go into quarantine units for each other. They described this professional support using expressions such as mutual respect and advocacy, intimate relationship, and comradeship. In the same vein, Brooks et al. (2019) also reported the importance of supportive relationships among healthcare workers exposed to disaster or crisis.

## Conclusion

This study investigated factors associated with PTG among HCWs who experienced the 2015 MERS outbreak in South Korea. A sequential, explanatory mixed-method design involving a quantitative survey, followed by qualitative interviews, was used to better understand this complicated psychosocial phenomenon. The quantitative analysis indicated that HCWs who experienced the MERS outbreak achieved PTG; the major factor associated with PTG was “resilience.” The qualitative interviews with seven nurses were analyzed using the deductive content analysis method based on the sub-factors of resilience. The analysis presented ample and rich data that correspond to the following sub-factors of resilience: hardiness, persistence, optimism, and support. Because the study's overall results show and illustrate how the participants developed PTG through the manifestation of resilience, it is not only important but necessary to help HCWs develop resilience in various ways and be aware of what can lead to resilience among HCWs.

The sense of professionalism, what motivated doctors and nurses to do what they do in the first place, played a central role in sustaining hardiness during the difficult times. That is, altruism, having compassion, and feelings of responsibility can strengthen their will to help the patients in need. However, the sense of responsibility and dedication cannot be forced or taken for granted, as it can also result in “feeling pressured or having resentment.” Hence, Rambaldini et al. (2005) discuss the relevant and important considerations when communicating with HCWs facing a crisis (2005): “acknowledging the unknown,” “providing support,” “ensuring collaboration,” etc.

Another essential aspect of resilience, persistence was observed when participants endured negative emotions and employed circumspect thinking. More specifically, participants echoed experiencing fear and enduring negative emotions associated with the uncertainty of the situation. Moreover, they become able to utilize coping skills and introspective reasoning to endure and persist during turbulent times. Therefore, it is crucial for HCWs to work through their negative emotions and employ circumspect thinking throughout the process of fighting infectious illnesses.

In addition, optimistic perspectives can play an essential role in promoting PTG as they can help HCWs attribute special meaning in their work and form a more positive attitude toward their practice and even future outbreaks. Lastly, developing a supportive and strong collegial relationship can promote resilience, leading to PTG as no other professionals understand what they are going through, being separated from the outside world and fighting for their patients' and their own lives in the face of an epidemic. Thus, a psychological support system to promote a strong collegial relationship among HCWs can not only foster resilience among HCWs but also increase opportunities for PTG.

These findings are meaningful in that they explain how HCWs achieve PTG through resilience explained by hardiness, persistence, optimism, and support, despite all the negative impacts of infectious illnesses such as MERS. More specifically, the results illustrate what issues or subjects need to be addressed when working with HCWs to promote cognitive restructuring and meaning-making, which are essential in developing PTG (Xu et al., 2016).

Moreover, this study findings convey important considerations for the healthcare field, as many HCWs are currently continuing the prolonged fight against the worldwide pandemic of COVID-19. Considering the potential adverse psychological effect of COVID-19 on HCWs, we must provide viable actions and strategies to address this issue, and help them understand their trauma and its impact on them. In return, it will improve the well-being and career longevity of HCWs (Okoli et al., 2021), and enhance the quality of the health care they provide.

Therefore, we strongly contend that there is a great need to expand systematic and aggressive measures to provide psychological and social support for HCWs who are helping patients exposed to infectious diseases. This would include the development and provision of programs encouraging peer support in addition to necessary supervision and training.

## Limitations and Implications for Future Research

This study also has its limitations. Firstly, participants were mostly homogeneous, as most of them were female nurses. Secondly, due to the previous findings explaining the relationship between PTG and resilience, the study employed sub-factors of resilience as a basis for qualitative analysis. Yet, it is also possible that there may be other factors associated with PTG among medical professionals. Thirdly, our sample size of 78 may be considered too small. However, the total number of HCWs who were eligible to participate in this study was less than 90 in the two hospitals where the study was conducted.

Perhaps this high level of participation of over 85% can be considered a strength of the study, as it represents the majority of HCWs exposed to MERS in one large metropolitan city in Korea. Another strength is that the study employed a sequential exploratory mixed-method design to investigate PTG of HCWs using both quantitative and qualitative lenses. Lastly, the study presents relevant information that many HCWs worldwide can relate to due to the COVID-19 crisis.

As for the implications for future research and practice, we suggest intervention studies that examine strategies for promoting intrapersonal and protective factors for resilience among HCWs. Also, further studies are needed to investigate systematic and departmental changes and actions required to promote the development of resilience and PTG among medical professionals. We hope that this will lead to the actual implementation of such measures to protect and support HCWs who are currently risking their lives at the frontline of fighting a worldwide pandemic.

## Data Availability Statement

All datasets generated in this study can be obtained from the authors with a request.

## Ethics Statement

The studies involving human participants were reviewed and approved by Institutional Review Board, Sangmyung University. The patients/participants provided their written informed consent to participate in this study.

## Author Contributions

MK and HH contributed to the development and design of the study. MK collected data. HH analyzed the quantitative data whereas MK and JL analyzed and interpreted the qualitative data. All authors wrote and proof-read the article, provided approval of the final version of the manuscript, and they agree to be accountable for all aspects of the content published in the study.

## Conflict of Interest

The authors declare that the research was conducted in the absence of any commercial or financial relationships that could be construed as a potential conflict of interest.

## Abbreviations

HCWs: Healthcare workers
PTG: Post-Traumatic Growth.
